# Secondary Breast Angiosarcoma Five Years After Radiation Therapy for Ductal Carcinoma In Situ

**DOI:** 10.7759/cureus.77954

**Published:** 2025-01-25

**Authors:** Abigayle Wyer, Mena Louis, Nathaniel Grabill, Pierpont Brown, Priscilla Strom

**Affiliations:** 1 Surgery, Northeast Georgia Medical Center Gainesville, Gainesville, USA; 2 General Surgery, Northeast Georgia Medical Center Gainesville, Gainesville, USA

**Keywords:** angiosarcoma of the breast, breast cancer, ductal carcinoma in situ, radiation induced angiosarcoma of the breast, radiation induced cancer, radiation therapy

## Abstract

Radiation-induced angiosarcoma (RIAS) of the breast is a rare and aggressive malignancy that emerges as a late complication of radiation therapy for primary breast cancer. We present the case of a 56-year-old woman with a history of high-grade ductal carcinoma in situ (DCIS) of the breast, previously treated with lumpectomy, sentinel lymph node biopsy, radiation therapy, and anastrozole. Five years after treatment, she developed new skin changes on her breast, including bruising and blistering following tanning bed use. Imaging studies showed diffuse skin thickening without discrete masses or lymphadenopathy. Biopsies confirmed the diagnosis of angiosarcoma. An extended mastectomy with wide surgical margins was performed. Pathological examination confirmed angiosarcoma with negative margins and no lymph node involvement. Additional excisions were undertaken to achieve 3 cm gross margins to ensure complete tumor removal due to the aggressive nature of the tumor. Patients presenting with new skin changes in previously irradiated areas, even many years after treatment, require careful evaluation. Nonspecific clinical and imaging findings necessitate prompt biopsy for accurate diagnosis. Surgical excision with wide margins remains the primary treatment modality, but standardized guidelines are lacking due to the rarity of RIAS. A multidisciplinary approach involving surgical oncology, pathology, radiology, and reconstructive surgery is essential for optimal patient management. Educating patients about potential late complications of radiation therapy and advising against additional risk factors, such as ultraviolet 2 exposure from tanning beds, are important. Further research is needed to establish evidence-based guidelines for the diagnosis, treatment, and follow-up of RIAS.

## Introduction

Radiation therapy has become an integral part of breast cancer treatment, substantially improving patient survival and reducing recurrence rates [1]. Despite its efficacy, radiation can infrequently lead to secondary malignancies, with radiation-induced angiosarcoma (RIAS) of the breast being one of the rarest and most aggressive forms [2]. This vascular tumor arises from the endothelial cells lining blood vessels and accounts for less than 0.05% of all breast cancers, making it a significant diagnostic and therapeutic challenge [3]. Typically developing five to 10 years after radiation exposure, RIAS often presents with subtle skin changes in the previously irradiated area [4]. Symptoms may include bruising, redness, or the appearance of a rash, which are easily mistaken for benign conditions or late effects of radiation therapy [5]. This nonspecific presentation can lead to delays in diagnosis, allowing the tumor to progress unchecked [6]. Due to its rarity and aggressive nature, there are no established standard treatment guidelines for RIAS of the breast [7]. Surgical resection with clear margins is considered the primary treatment, but achieving negative margins is difficult because of the tumor's infiltrative growth pattern [8]. The effectiveness of adjuvant therapies like chemotherapy and additional radiation remains uncertain, and decisions are often made on an individual basis [9].

## Case presentation

**Figure 1:**
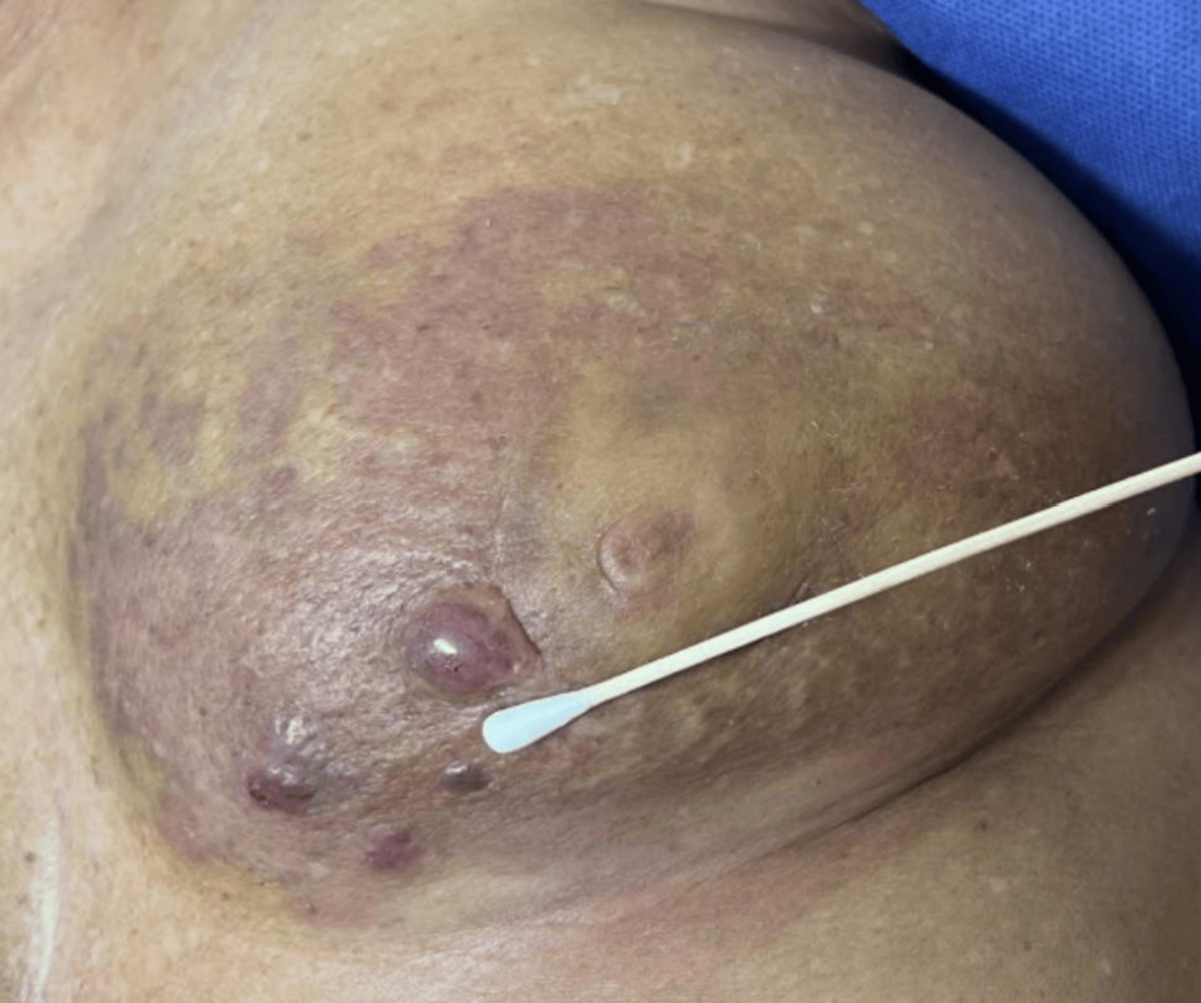
Pre-operative photo of the left breast with skin changes, including bruising and blisters.

A 56-year-old woman with a medical history of hypertension, migraines, depression, and previously treated ductal carcinoma in situ (DCIS) of the left breast presented with new skin changes on her left breast, including bruising and blisters. The initial DCIS was high-grade with comedonecrosis, estrogen receptor-positive, and progesterone receptor-negative. She underwent a lumpectomy with sentinel lymph node biopsy (SLNB) five years prior, which revealed no lymph node involvement. Postoperative treatment included radiation therapy to the left breast and initiation of anastrozole. She maintained regular follow-up with mammograms every six months for two years, then annually. While on anastrozole therapy, she noticed bruising on her left breast, which she initially attributed to minor trauma. Later, she developed blisters in the same area after using a tanning bed (Figure 1). Concerned about these skin changes, she sought medical evaluation.

**Figure 2:**
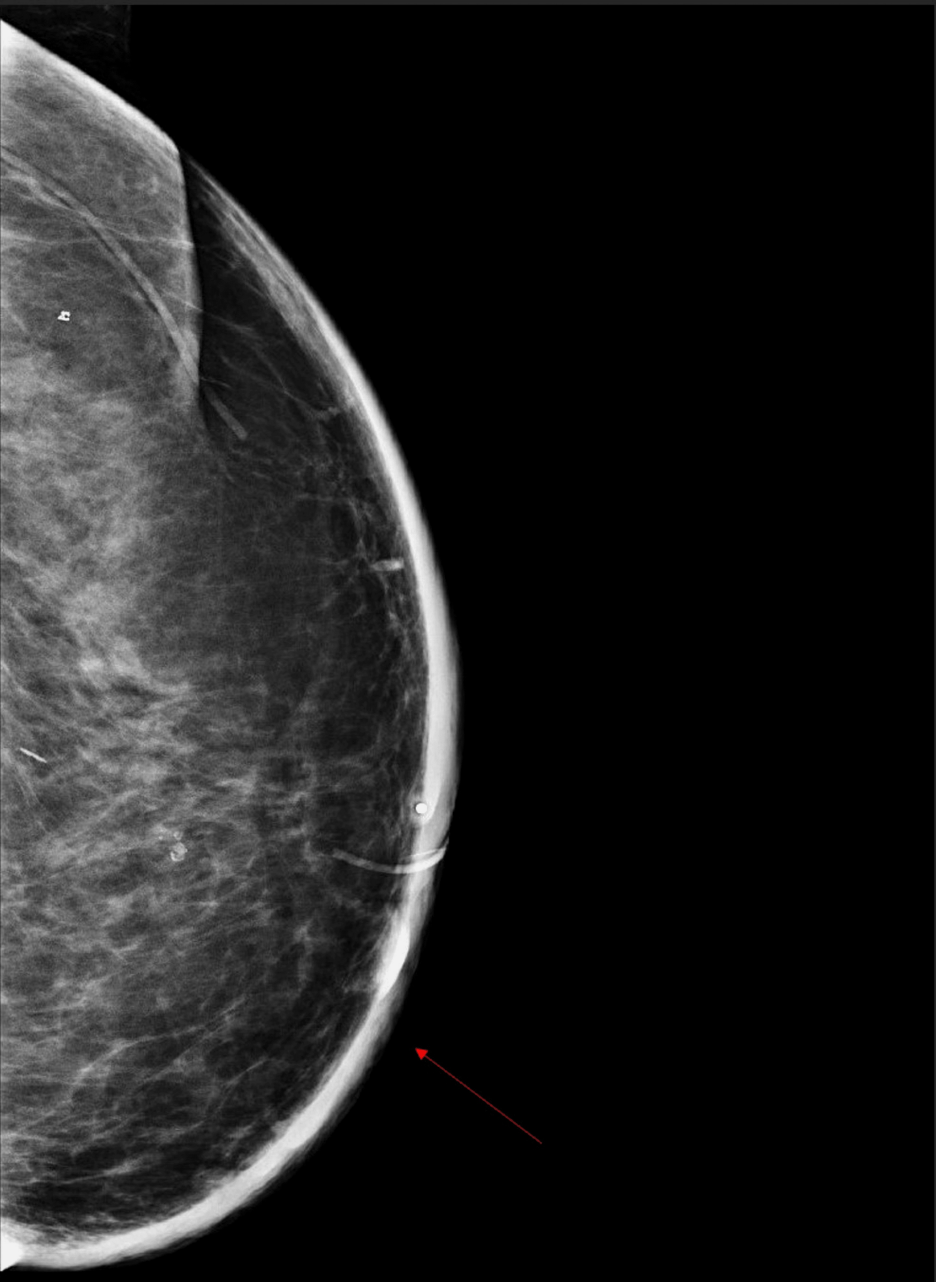
Diagnostic mammogram of the left breast (cranial-caudal view) demonstrating diffuse skin thickening (red arrow) without changes in the breast parenchyma.

**Figure 3:**
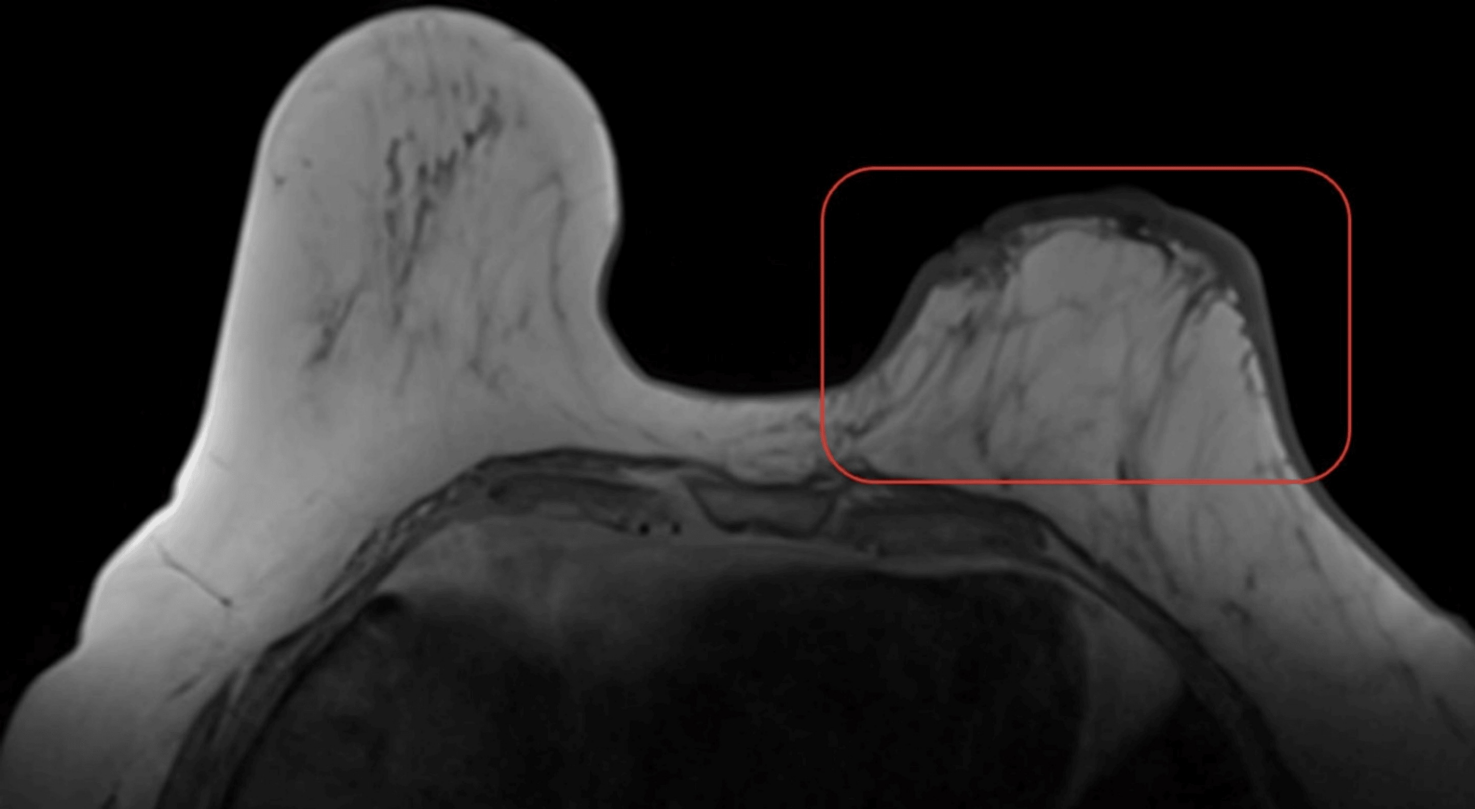
Magnetic resonance imaging (T1-weighted phase, axial view) revealed diffuse skin thickening of the left breast (red box) with increased thickness and nodularity around the nipple area with abnormal parenchymal enhancement just beneath the skin surface.

A diagnostic mammogram of the left breast showed diffuse skin thickening without changes in the breast parenchyma or signs of lymphadenopathy (Figure 2). Magnetic resonance imaging (MRI) revealed diffuse skin thickening with increased thickness and nodularity around the nipple area. There was abnormal parenchymal enhancement just beneath the skin surface, with multiple small discrete nodules within the skin, some connected to the nipple (Figure 3). No suspicious lymph nodes were detected in the axillary or internal mammary regions.

**Figure 4:**
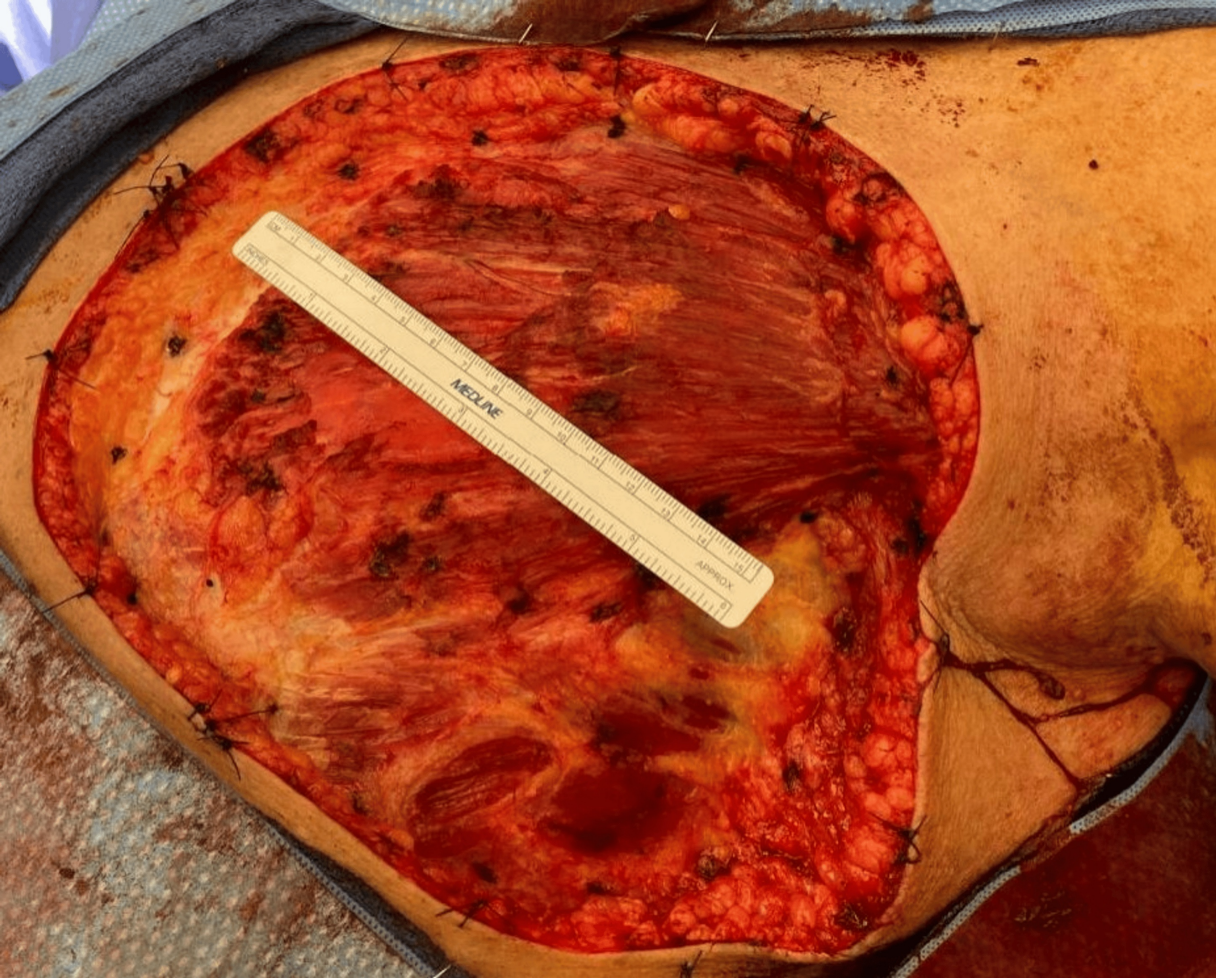
Intra-operative photo of the left breast with 3-cm margins resulting in a large wound extending to the clavicle, across to the medial aspect of the contralateral breast, and onto the abdominal wall.

A punch biopsy was performed, which revealed a vascular lesion that appeared to be a hemangioma; however, it had focal atypical features, and an incisional biopsy was recommended. It was followed by an incisional biopsy, which confirmed angiosarcoma of the left breast. The case was discussed at a multidisciplinary tumor board meeting, and the consensus was to proceed with surgery aiming for clear margins. Prior to surgery, a CT of the chest/abdomen/pelvis was performed, which revealed no evidence of metastatic disease. She underwent a left simple mastectomy with the application of a biodegradable temporizing matrix and wound vacuum-assisted closure (VAC). During surgery, 3-cm margins were taken circumferentially, extending up to the clavicle, across to the medial aspect of the contralateral breast, and onto the abdominal wall (Figure 4). Portions of the underlying pectoralis major muscle were also removed due to proximity to the most involved area. The surgical specimen was meticulously oriented for margin assessment.

**Figure 5:**
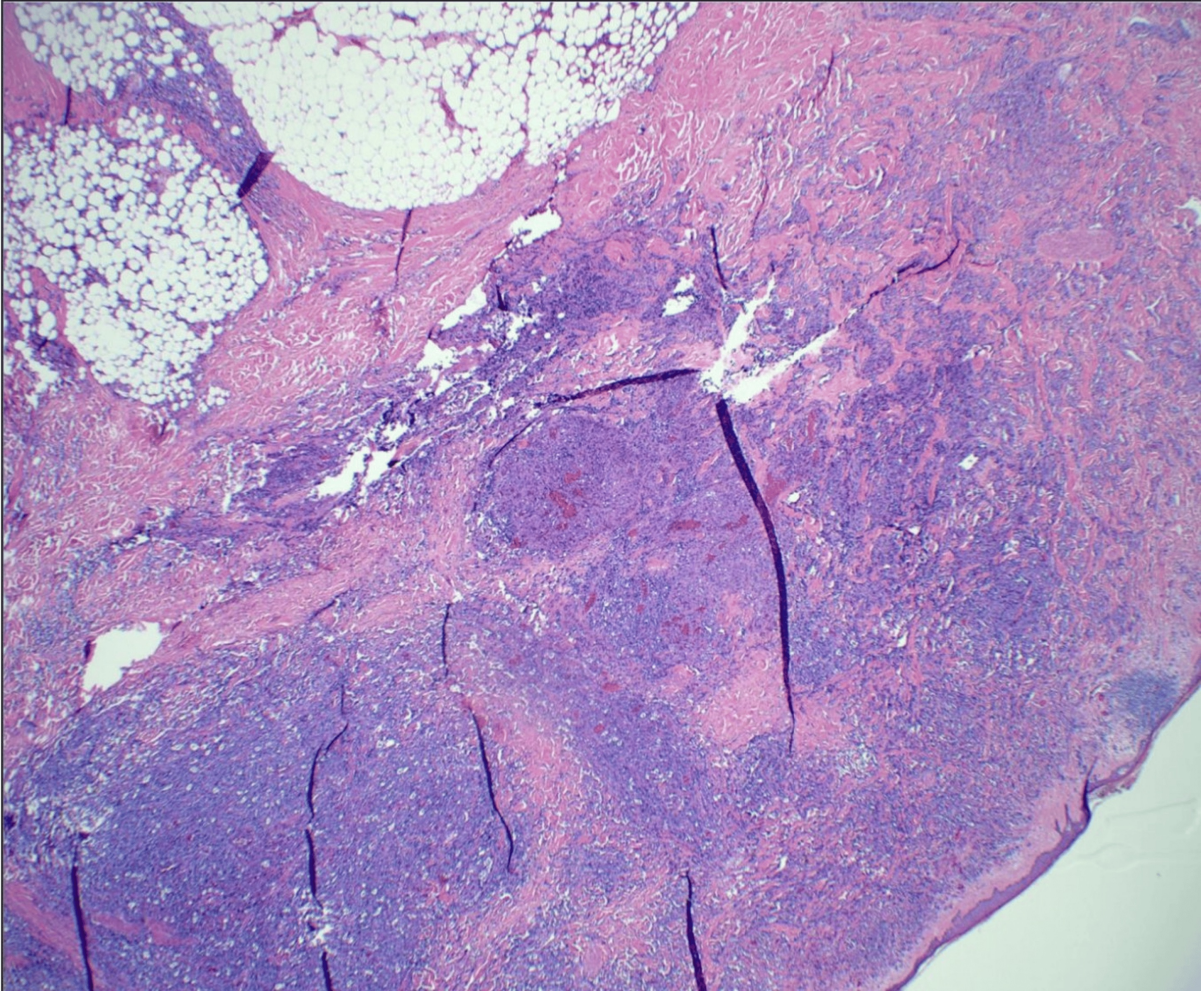
Histopathology slide with H&E stain at 20× magnification showing the angiosarcoma extending down to subcutaneous fat with the tumor extending down into breast parenchyma.

**Figure 6:**
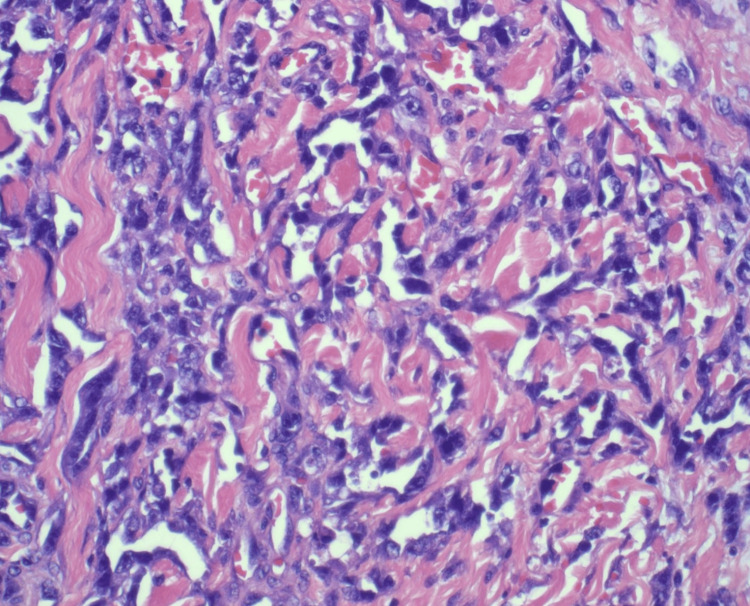
Histopathology slide with H&E stain at 400× magnification showing tumor cells with irregular, angulated, and variably dilated vascular channels, often with a sieve-like morphology called a "vasoformative pattern." The cells are ovoid to angulated with hyperchromatic nuclei.

**Figure 7:**
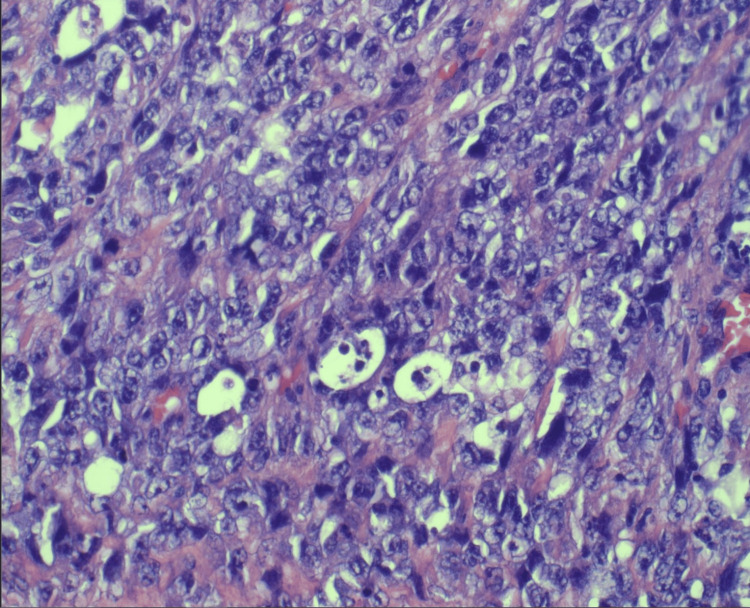
Histopathology slide with H&E stain at 400× magnification showing a poorly differentiated portion of the tumor with more solid areas. The cells are larger, with occasional distinct nucleoli and increased mitosis and apoptotic cells.

Pathological examination confirmed angiosarcoma with negative margins. A small amount of vascular atypia was noted at specific margins, but these areas were not diagnostic of angiosarcoma and could represent reactive changes to prior radiation therapy (Figures 5-7). Due to these irregularities, she was taken back to the operating room, and additional excisions were performed in these areas to ensure complete removal. No angiosarcoma or atypical vascular proliferation was found in the re-excised specimens. No immunohistochemistry was performed. One lymph node was sampled and found to be negative for tumor involvement.

Postoperative laboratory values, including white blood cell count and hemoglobin, remained within acceptable ranges. The patient was managed with wound care, including adjustments to the biodegradable matrix and wound VAC. Plans for further reconstructive surgery were considered pending confirmation of negative margins and adequate healing. She will undergo close follow-up with imaging three months after surgery.

## Discussion

RIAS of the breast is an exceptionally rare and aggressive malignancy that arises as a late complication of radiation therapy administered for primary breast cancer [10]. It represents less than 0.05% of all breast cancers and poses significant diagnostic and therapeutic challenges due to its rarity and nonspecific presentation [11]. In this case, the patient developed angiosarcoma approximately five years after receiving radiation therapy for high-grade DCIS of the left breast. The latency period for RIAS typically ranges from five to 10 years, which aligns with the timeline observed here [12]. The relatively short latency emphasizes the need for vigilance in monitoring patients who have undergone radiation therapy, even within a few years post-treatment [13].

The patient initially noticed bruising and later developed blisters on the left breast after using a tanning bed. These skin changes were subtle and could easily be mistaken for benign conditions such as dermatitis or radiation recall reactions [14]. The use of a tanning bed, which exposes the skin to ultraviolet (UV) radiation, may have exacerbated the skin changes, although a direct causal relationship with angiosarcoma is not established [15].

Imaging studies, including mammography and MRI, revealed diffuse skin thickening and nodularity without discrete masses or lymphadenopathy. These findings are nonspecific and can be associated with a range of benign and malignant conditions. In angiosarcoma, imaging often lacks distinctive features, making histopathological examination crucial for diagnosis [16]. A punch biopsy followed by an incisional biopsy was necessary to obtain adequate tissue for a definitive diagnosis. Histologically, angiosarcoma is characterized by atypical, irregular vascular channels lined by malignant endothelial cells. In this case, the initial biopsy showed atypical 11 vascular proliferation extending to the margins, prompting further surgical intervention to achieve clear margins [17].

RIAS arises from the malignant transformation of endothelial cells in the irradiated field [18]. The exact pathogenesis involves radiation-induced DNA damage leading to genetic mutations and chromosomal aberrations [19]. Risk factors for developing RIAS include higher doses of radiation, younger age at the time of radiation therapy, and the presence of lymphedema [20]. While the patient's use of a tanning bed introduced additional UV exposure, its role in the development of angiosarcoma remains speculative [20]. Nonetheless, UV radiation is a known risk factor for other skin malignancies and may contribute to cumulative DNA damage [15]. In addition, vinyl chloride exposure is a known risk factor for the development of angiosarcoma. 

The mainstay of treatment for RIAS is surgical excision with wide margins to reduce the risk of local recurrence [12]. In this case, the patient underwent an extended left mastectomy with 3-cm margins, including portions of the underlying pectoralis major muscle. Achieving negative margins is often challenging due to the infiltrative and multifocal nature of angiosarcoma [20]. The use of a biodegradable temporizing matrix and wound VAC facilitated wound management and may improve healing outcomes [18]. The decision to perform additional excisions for areas with vascular atypia was essential for thorough surgical clearance. The role of adjuvant therapies, such as chemotherapy and additional radiation, is not well established for RIAS [20]. Some studies suggest potential benefits from taxane-based chemotherapy, but evidence is limited due to the rarity of the condition [20]. Re-irradiation is generally avoided because the tumor arises in previously irradiated tissue.

The necessity of SLNB in angiosarcoma remains controversial [4]. Angiosarcoma primarily metastasizes hematogenously rather than through lymphatic channels, and lymph node involvement is uncommon [12]. In this patient, one lymph node was sampled and found to be negative for tumor involvement. The decision to perform SLNB may be guided by individual patient factors and institutional protocols, but routine SLNB is not universally recommended for cutaneous angiosarcoma of the breast.

RIAS carries a poor prognosis due to its aggressive behavior and high rates of local recurrence and distant metastasis [18]. Factors influencing prognosis include tumor size, depth of invasion, margin status, and the ability to achieve complete surgical excision [3]. However, there is currently no standard protocol for surgery. Close postoperative surveillance is essential for early detection of recurrence. Follow-up protocols may include regular physical examinations and imaging studies, although the optimal surveillance strategy is not well-defined [19].

Managing RIAS requires a multidisciplinary team involving surgical oncologists, plastic and reconstructive surgeons, pathologists, radiologists, and medical oncologists. Collaborative care ensures comprehensive treatment planning, addressing surgical challenges, wound management, and consideration of adjuvant therapies. In this case, the consensus of the tumor board guided the surgical approach, and plastic surgery expertise was crucial for wound coverage and planning potential reconstruction.

Literature reports on RIAS are limited to case reports and small case series due to its rarity [10]. Studies consistently highlight the difficulty in early diagnosis, the challenges in achieving clear 13 surgical margins, and the limited efficacy of adjuvant therapies [3]. The patient's presentation, diagnostic journey, and management align with reported cases. Nascimento et al. reported high recurrence rates despite aggressive surgical interventions, emphasizing the need for early detection and wide excision [20], and discussed the poor prognosis associated with RIAS and the lack of standardized treatment protocols [20].

## Conclusions

Educational implications of this case include raising awareness of RIAS, the importance of biopsies, surgical challenges, patient education, and the need for research. Clinicians should maintain a high index of suspicion for angiosarcoma in patients presenting with new skin changes in previously irradiated areas, even several years after therapy. Early biopsy of suspicious skin lesions is essential for prompt diagnosis. Imaging alone may not provide definitive information. Achieving negative margins requires meticulous surgical planning and may involve extensive resections. Collaboration with reconstructive surgeons is important for managing large defects. Patients should be informed about the potential long-term risks of radiation therapy, including the rare possibility of secondary malignancies. Advising against additional UV exposure, such as tanning bed use, is prudent. The rarity of RIAS highlights the need for continued research to establish evidence-based guidelines for diagnosis, treatment, and follow-up.

RIAS of the breast presents significant diagnostic and therapeutic challenges due to its rarity and aggressive nature. Early recognition through prompt evaluation and biopsy of new skin changes in previously irradiated areas is essential. Surgical excision with wide 14 margins remains the primary treatment, necessitating meticulous planning and a multidisciplinary approach. Educating patients about the long-term risks of radiation therapy and advising against additional UV exposure can aid in early detection and prevention.
